# A comparison between neurological clinical signs, cerebrospinal fluid analysis, cross-sectional CNS imaging, and infectious disease testing in 168 dogs with infectious or immune-mediated meningoencephalomyelitis from Brazil

**DOI:** 10.3389/fvets.2023.1239106

**Published:** 2023-10-25

**Authors:** Fernando Swiech Bach, Carolyn Cray, Ana Paula Burgos, José Ademar Villanova Junior, Fabiano Montiani-Ferreira

**Affiliations:** ^1^Neurology Service, Clinivet Veterinary Hospital, Curitiba, Brazil; ^2^Division of Comparative Pathology, Department of Pathology and Laboratory Medicine, University of Miami Miller School of Medicine, Miami, FL, United States; ^3^Small Animal Surgery Service, Catholic University of Paraná, Curitiba, Brazil; ^4^Comparative Ophthalmology Lab (LABOCO), Federal University of Paraná, Curitiba, Brazil

**Keywords:** meningoencephalomyelitis, meningoencephalitis, dogs, autoimmune, infection, cerebral spinal fluid, neurological panel

## Abstract

This retrospective study evaluated canine patients with presumptively diagnosed meningoencephalomyelitis (ME) based on neurological clinical signs, cerebrospinal fluid (CSF) analysis, cross-sectional imaging, and infectious disease testing with a limited neurological-focused polymerase chain reaction (PCR) panel performed on blood and CSF. The first goal was to determine the proportion of dogs where the condition was caused by an infectious agent versus a probable immune-mediated etiology (i.e., meningoencephalomyelitis of unknown origin; MUO) in our geographic region. The secondary goals of this study were to examine and define associations between abnormal CSF test results and cross-sectional neuroimaging findings, in addition to defining the age and most common neurological clinical signs in each group of ME. A total of 168 dogs matched the inclusion criteria with magnetic resonance imaging (MRI) performed in 130 dogs and computed tomography (CT) performed in 38 dogs. Presumptive MUO was observed in 152/168 (90.5%) of dogs and infectious ME was identified in 16/168 (9.5%) of dogs (*p* < 0.0001). Canine distemper virus (CDV) was the most common cause of infectious ME in 10/16 dogs (62.5%). Of the total cases with a positive infectious disease result, 3/16 (18.7%) had normal CSF results and 13/16 (81.3%) had abnormal CSF results (*p* = 0.0078). MRI and CT abnormalities in the brain were detected in 74 and 39% of dogs with inflammatory CSF, respectively. MRI and CT abnormalities in the spinal cord were detected in 90 and 57% of dogs with inflammatory CSF results, respectively. Age was not significantly different between infectious ME and presumptive MUO groups (*p* = 0.15). Seizures were the most common clinical sign reported for both MUO (36.8% of cases) and infectious ME (31.2% of cases). In conclusion, presumptive MUO is significantly more common than infectious ME in this population of dogs. Furthermore, although normal CSF results were uncommon in dogs with infectious ME, this finding occurred in several patients (3/16), suggesting that infectious disease testing should be considered even in the face of normal CSF results. Finally, MRI was more sensitive than CT in the detection of abnormalities when dogs with ME had inflammatory CSF results but was not 100% sensitive, suggesting CSF analysis should be performed to rule out inflammation even when no abnormalities are detected on MRI or CT.

## 1. Introduction

Meningoencephalomyelitis (ME) can be defined as inflammation of the meninges (pia mater, arachnoid, and dura mater), brain, and spinal cord parenchyma (1). The etiology of ME may be divided into two general categories: (1) infectious diseases (i.e., bacterial, viral, protozoal, fungal, or parasitic etiologies) and (2) non-infectious (i.e., immune-mediated). The latter is the most common in dogs and is broadly categorized as meningoencephalomyelitis of unknown origin (MUO) (2). Although definitive diagnosis of MUO is through histopathology (2), antemortem diagnosis is supported by cross-sectional imaging of the central nervous system (CNS), cerebrospinal fluid (CSF) analysis, and lack of identifiable infectious agents. The etiopathogenesis of MUO is debatable but investigations geared to define an infectious etiology have failed to reveal a consistent infectious agent (3). In addition, patient response to immunosuppression is supportive of immune-mediated etiopathogenesis (2).

Neurologic clinical signs of ME depend on localization within CNS and severity (4). The most common clinical signs observed in a study of 94 dogs diagnosed with MUO were abnormal mentation or behavior, proprioceptive deficits, cranial nerve deficits, ataxia, and seizure activity (5). The clinical signs in infectious-related ME will depend on the infectious agent, however, seizures, ataxia, cranial nerve deficits, and proprioceptive deficits can also be observed (2). It should be noted that the clinical signs of CNS inflammatory disorders are frequently very similar to those of infectious CNS diseases and even those of neoplasia (2).

Diagnosis usually is based on advanced imaging (CT or MRI), cerebrospinal fluid (CSF) analysis, and antibody and antigen tests to rule out an infectious disease. The correlation regarding inflammatory CSF results and MRI change findings was previously described in the study by Lamb et al. who reported that inflammatory CSF results were associated with normal MRI results in 24% of the cases. In neuroimaging, most cases of neoplastic lesions, which are generally unifocal, are differentiated from inflammatory diseases, which are usually multifocal (2). Therefore, the major differential diagnostic decision is between infectious and noninfectious ME. In most European countries and the United States, noninfectious inflammatory diseases of the CNS, which can affect the brain, spinal cord, and/or the meninges, are much more common than infectious diseases (2). As definitive diagnosis of MUO requires histopathological analysis, for a presumptive diagnosis, a multimodal approach is needed (2). Although magnetic resonance imaging (MRI) can identify abnormalities consistent with inflammatory disease in the brain or spinal cord, it is not always possible to obtain a definitive diagnosis. Thus, for antemortem diagnosis of MUO or infectious ME, CSF analysis and testing for infectious diseases commonly affecting the CNS in the geographic region should be used (2). For the latter, this can often be accomplished through the use of a neurological focused infectious disease PCR panel. CSF analysis in MUO patients usually shows mononuclear pleocytosis and high protein concentration and can vary considerably in relationship to case severity (5, 6). The CSF of infection-based ME will depend on the infectious agent. For example, cases of bacterial infection are characterized by neutrophilic/degenerative pleocytosis, high protein, and low glucose. In contrast, viral infection usually is observed with pleocytosis lymphocytes and high protein. Protozoal infection can be associated with eosinophil pleocytosis and high protein (7).

Differentiating infectious vs. non-infectious ME in the clinical patient is important (6). The prognosis for dogs with MUO is variable, survival interval range of 1–1,200 days (6). In one study, it was estimated that 56% of dogs with a presumptive MUO diagnosis had a survival interval of 0 to 52 days after the start of treatment (8). Therefore, aggressive therapies with immunosuppressive doses of corticosteroids before a conclusive diagnosis of non-infectious disease can be justified because of the high mortality rate and short survival time (8). The immunosuppressive treatment should start as soon as possible in MUO, although, it is important to rule out the infectious disease using serology and PCR based infectious disease tests (6). However, extended turnaround times of these tests can make the treatment decision challenging, because of aggressive immunosuppression, which can be fatal in the case of an infectious etiology (9). Other immunosuppressive drugs besides corticosteroids have also been described in some MUO studies (10–14). While the factors contributing to prognosis are not well defined, the progression of the disease, neuroimaging findings, grade of inflammation in CSF analysis, and the severity of the clinical signs are considered prognosis indicators (8).

In the present study, the medical records of 168 dogs with presumptive meningoencephalomyelitis of unknown origin (MUO) and infectious ME were retrospectively evaluated. Geolocation may influence the incidence of etiologies of ME (2). To this point, in Brazil, a previous study supported canine distemper virus (CDV) related to ME as a common diagnosis (15). Thus, the primary aim of this study was to evaluate the incidence of ME caused by infectious diseases or MUO in Curitiba, Paraná, Brazil. Our primary hypothesis was that Non-infectious (i.e., immune-mediated) ME are more common than infectious ME in Brazil. The secondary aims were to examine the relationship between neuroimaging findings versus CSF analysis as well as the definition of the most common neurologic clinical signs and age of animals in each group of ME. Our secondary questions included: (a) is it possible that inflammatory CSF results have normal neuroimaging and abnormal neuroimaging can have normal CSF results? (b) Are seizures the most common clinical sign in cases of ME? And finally, (c) does infectious ME affect young dogs more than immune-mediated ME? It is expected the answers to these lines of investigation will aid clinicians in the interpretation of results and diagnosis of canine ME.

## 2. Materials and methods

### 2.1. Ethics committee

The present animal study was approved and reviewed by the Ethics Committee of the Pontifical Catholic University of Paraná (PUCPR, Brazil). Written informed consent was obtained from the hospital owners for the participation of their animals in this study.

### 2.2. Kind of study

Retrospective, transversal, and non-randomized.

### 2.3. Inclusion criteria

Data from a total of 168 dogs with CSF samples and neurological focused infectious disease PCR panel from 2015 to 2022 that were referred to a veterinary neurology service in a private veterinary hospital in Curitiba (Clinivet), Paraná, Brazil.

All cases needed to fulfill the following criteria: detailed neurological exam; recorded neurological clinical signs; blood work including complete blood count, ALT, creatine, urea, albumin, glucose, ammonia; cerebrospinal fluid (CSF) analysis; cross sectional images; and neurological infectious disease panel (referred to going forward as PCR panel). The latter test is conducted by Idexx Laboratories (Wesbrook, Maine) and includes the following: *Bartonella* spp.*, Borrelia burgdorfi, Blastomyces dermatitidis*, CDV, *Coccidioides* spp.*, Cryptococcus* spp.*, Histoplasma capsulatum, Neospora* spp.*, Toxoplasma gondii* and West Nile Virus. All samples for the PCR panel were performed using blood and CSF samples and results were provided as positive or negative.

**Figure 1:**
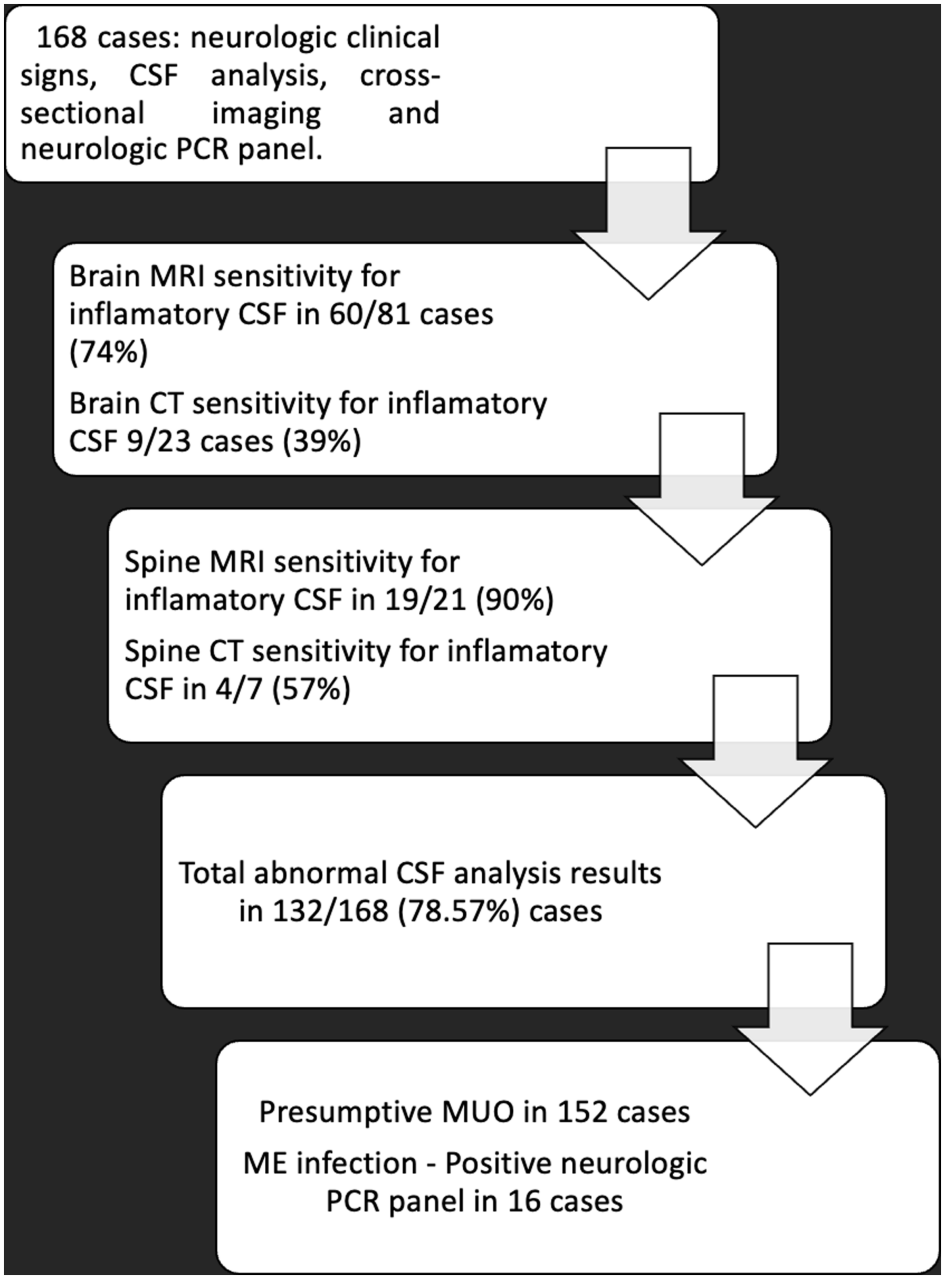
Flowchart demonstrating main results of the study.

Figure 1 presents a flow chart depicting the sequence of the study and the main results.

### 2.4. Anesthesia and CSF collection and analysis

All dogs were anesthetized using intravenous bolus of propofol (5 mg/kg) and intubated with a tracheal tube. The same person (FB) performed all CSF collections at the cisterna magna, using the technique described in the literature (16). The patient was positioned in lateral recumbency, and the neck flexed until a 90 angle between the nasal bone and cervical spine was formed. The puncture area was surgically prepared by clipping the hair. A sterile spinal needle was used to collect CSF and immediately sent to the laboratory for analysis. CSF results were evaluated by the following: number of nucleated cells, erythrocytes, lymphocytes, neutrophils (segmented or degenerated), monocytes, eosinophils; protein and glucose concentrations; and presence or absence of microorganisms. CSF cultures were not performed. The CSF samples were collected immediately after the CT or MRI procedures.

CSF results were considered normal if there was <5 nucleated cells and no abnormal distribution of white cells, protein concentration < 30 mg/dL, 70% of serum glucose, and absence of microorganism in the sample.

### 2.5. Neuroimaging techniques

MRI testing (1.5 Tesla, Avanto, Siemens, Erlangen, Germany) was performed with patients also anesthetized with intravenous propofol (B. Braun, Melsungen, Germany) at 5 mg/kg. These patients were intubated and placed in dorsal recumbency. Pulse sequences for the brain exams included, T2 sagittal plane, T1-weighted, T2-weighted, and FLAIR in transversal planes, Diffusion, ADC, and SWI in transverse planes, plus T1-weighted with fat suppression after administration of intravenous paramagnetic contrast medium (gadoteridol – 0.5 mmoL/mL, ProHance Bracco imaging, Germany) at a dose of 0.1 mmol/kg. For the spinal images pulse sequences included were sagittal plane T1-weighted, T2-weighted, HASTE and STIR, transverse plane T2-weighted, dorsal plane STIR and sagittal/transverse plane post-contrast T1-weighted with fat suppression after administration of intravenous paramagnetic contrast medium at the same dose that in brain exams. All MRI exams were performed in a human hospital.

The CT scans (Somatom Spirit multislice, Siemens, Munich, Germany) were performed with the patients under sedation using intravenous dexmedetomidine (Zoetis, São Paulo-SP, Brazil) 10 mcg/kg and placed in dorsal recumbency, CT exams were performed without contrast and after intravenous contrast iohexol (Omnipaque 300, GE Healthcare, Barueri, SP, Brazil) at a dose of 1.5 mL/kg, injected intravenous. All CT exams were performed at Clinivet Veterinary Hospital.

To avoid interobserver discrepancies, all MRI and CT images were prospectively evaluated by the same examiner (FB) who has more than 15 years of clinical experience in neurology, neuro-imaging studies, and neurosurgery.

Dogs identified with solitary mass which was attached in the meninges with a dural tail which strongly suggested a diagnosis of meningioma were excluded. Dogs with neuroimaging that suggested vascular disease (ischemic) showing restriction on diffusion images and hyposignal on ADC images were also excluded. In addition, dogs were excluded with otitis media and communication with brainstem, due to a lack of PCR results in those cases.

Cases with CT included 38 dogs and cases with MRI included 130 dogs.

### 2.6. Criteria used to consider presumptive MUO

The indicators used for the diagnosis of dogs with MUO were neurological clinical signs, negative infectious disease panel results, and CSF analysis showing an increase in nucleated cells (>5 μL) with at least >50% lymphocytic/monocytic pleocytosis and/or protein (>30 mg/dL) (6).

**Figure 2:**
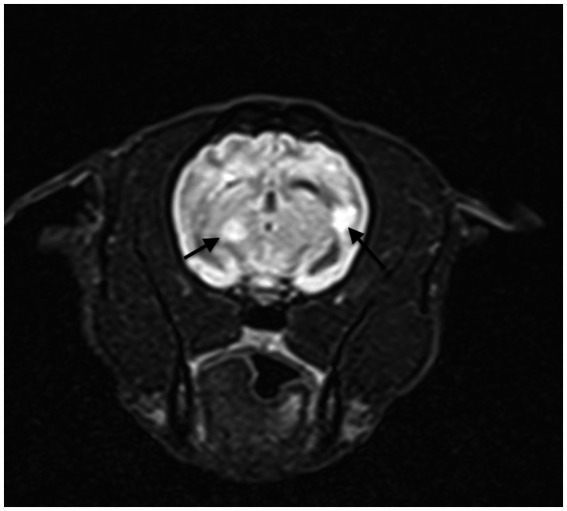
Transversal, FLAIR T2 weighted MRI showing hyperintesity in multifocal lesions suggestive of auto-immune ME.

Neuroimaging with changes inclusive of the following was also part of this criteria: MRI cases that showed multifocal hyperintensity lesions on T2 or FLAIR, with or without contrast enhancement were also indicators (6) (Figure 2). CT also showed multiple hypoattenuating or hyperattenuating lesions with or without contrast enhancement.

Indicators for considering presumptive MUO as a diagnosis in a few dogs with normal CSF analysis and normal neuroimaging were as follows: neurological clinical signs, breed, and partial or full response to immunosuppressive therapy.

### 2.7. Criteria used for ME of infectious origin

The indicators used for ME of infectious origin were positive results in the infectious disease PCR panel in the blood sample and CSF.

### 2.8. Data analysis

#### 2.8.1. Neurologic PCR panel and CSF analysis

The following groups were compared by statistical analysis: Positive PCR vs. normal CSF results. Positive (PCR vs. abnormal CSF results. Negative PCR vs. Abnormal CSF. Negative PCR vs. Normal CSF.

#### 2.8.2. Correlation neuroimaging and CSF analysis

Cases with computed tomography (CT) included 38 dogs and cases with magnetic resonance imaging (MRI) included 130 dogs.

All intracranial and spinal cord MRI cases were analyzed and compared in this manner: the total MRI performed in CSF normal cases, and the total MRI performed in CSF abnormal cases. MRI results (normal or abnormal) in patients with a normal CSF analysis; and MRI results (normal and abnormal) in patients with abnormal CSF analysis (Supplementray Table 2). MRI findings in dogs with neurological panel positive were also compared.

All intracranial and spinal cord CT cases were analyzed and compared in this manner: The total CT performed in CSF normal cases, and the total CT performed in CSF abnormal cases. CT results (normal or abnormal) in patients with a normal CSF analysis; CT results (normal and abnormal) in patients with an abnormal CSF analysis (Supplementray Table 3).

### 2.9. Statistical analysis

Results were organized in contingency tables. Sensitivity and specificity were calculated (brain and spine MRI, CT scan, and abnormal CSF results). The following statistical tests were applied: Chi-square test (presumptive MUO cases versus cases diagnosed as infectious ME); and Fisher’s exact test (for PCR-positive results for infectious agents versus CSF results). Mann–Whitney test (comparing the non-normally distributed age data in the presumptive MUO versus infectious ME groups). The confidence interval (CI) was 95% and *p*-values ≤0.05 were considered significant. The software MedCalc^®^ Statistical Software version 20.027 (MedCalc Software Ltd., Ostend, Belgium) was used for these calculations.

## 3. Results

A total of 168 cases were analyzed including 59.2% females and 40.8% males. The mean age was 5 years (range 2 months to 15 years). In the MUO group, the mean age was 5.5 years (4 months - 11 years), and in the ME infection group, the mean age was 4.5 years (2 months - 15 years). This difference was not significant (*p* = 0.15). Among the 168 dogs in the present study, 30 breeds were included, and the two most affected breeds were mixed breed dogs (28%) and the Maltese (13%) (Supplementray Table 1).

### 3.1. CSF and PCR panel results

Of the 168 CSF samples, a total of 152 dogs (90.5%) were diagnosed with presumptive MUO (probable immune-mediated etiology), and 16 dogs (9.5%) were diagnosed with infectious ME; this difference was significant (*p* < 0.0001). In the MUO group, 119/152 dogs had abnormal CSF results (78.3%), and 33/152 dogs had normal CSF results (21.7%). In the infectious ME group, the CSF analysis showed abnormal results in 13/16 (81.3%) versus 3/16 (18.7%), with normal CSF analysis. In addition, the presence of abnormal CSF results were significantly correlated with positive results in the infectious disease PCR panel (*p* = 0.0078), 95% CI of odds ratio: 0.025 to 0.728.

Four different infectious agents were diagnosed by the infectious disease PCR panel: 1) CDV 10/16 (62.5%); 2) *Toxoplasma gondii* 2/16 (12.5%); 3) *Neospora* sp. Plus *Toxoplasma gondii* 1/16 (6.3%); 4) *Borrelia burgdorferi* 3/16 (18.7%).

Of the CDV cases, 7/10 (70%) showed abnormal CSF analysis. Interestingly, the predominant cell type observed in the CDV cases were > 50% lymphocytes and, in toxoplasmosis and neosporosis cases, more than 50% neutrophils were observed.

Of the 168 dogs examined in this study, 132/168 (78.6%) had abnormal CSF analysis, and 36/168 (21.4%) were considered normal. Of the 132 total abnormal CSF results 92 dogs had increased nucleated cells >5 mm3 with or without accompanying elevated protein (>30 mg/dL) in their respective CSF samples and 71 dogs had elevated protein with or without accompanying increased nucleated cells. In those samples with abnormal CSF results, the mean number of nucleated cells was 108.25 (varying from 6 to 2,224 nucleated cells). The mean concentration of protein was 67.05 mg/dL and (range 31 to 720 mg/dL). No samples with microorganisms were detected directly using CSF microscopy. Regarding the samples with abnormal CSF analysis, 119/132 (90.2%) had negative results for the infectious disease PCR panel and 13/132 (9.8%) had a positive result. For the samples with normal CSF analysis, 33/36 (91.7%) had negative PCR panel results and three normal CSF analysis samples 3/36 (8.3%) were positive for CDV per the PCR panel.

### 3.2. Neuroimaging and CSF results

A total of 130 MRI (106 brain, 14 cervical, 10 thoracolumbar) and 38 CT scans (30 brain, 4 cervical, and 4 thoracolumbar) were performed. Of all 168 neuroimages, the spinal cord was examined in 32/168 cases (19.0%).

#### 3.2.1. MRI and CSF results

The total number of brain MRI analyzed was 106 cases. The relative sensitivity value was 74% (95% CI: 63.1 to 83.1%) and specificity was 64% (95% CI: 42.5 to 82%), for the detection of changes in dogs with CSF results supportive of inflammation. A comparison of results: neuroimaging analysis versus results from CSF analysis is presented in Supplementray Table 2. Five (50%) of ten distemper cases were found to be abnormal by MRI.

Regarding myelopathies: The total number of spinal cord MRI analyzed included 24 cases. The relative sensitivity value was 90.5% (95% CI: 69.7 to 98.8%) and specificity was 66.9% (95% CI: 9.4 to 99.2%) for spinal cord MRI detected inflammatory changes in CSF results. A comparison of results found in the MRI neuroimaging analysis versus results from CSF analysis is presented in Supplementray Table 3.

**Figure 3:**
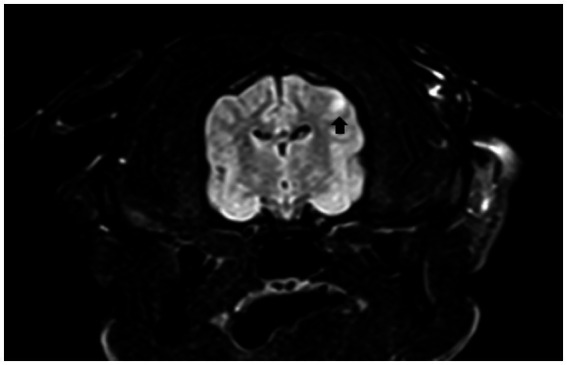
Transversal, FLAIR T2 weighted MRI showing hyperintensity lesion.

Of all cases where the brain was examined by MRI, the most common abnormal findings were multiple lesions with hyperintensity on T2 and FLAIR sequences with or without contrast enhancement in the meninges and/or in the lesions (Figures 2, 3). Of all cases where the spinal cord was examined, the most common abnormal finding were multiple lesions with hyperintensity on T2 and SWI sequences with or without contrast enhancement in the meninges and/or in the lesions.

One representative brain MRI lesion is shown in Figure 2.

#### 3.2.2. CT and CSF results

Brain CT results were available in 30 cases. The relative sensitivity value was 39.1% (95% CI: 19.7 to 61.4%) and specificity was 71.4% (95% CI: 29 to 96.3%) for brain CT, in the detection of changes in cases with inflammatory CSF results A comparison of results found in the brain CT neuroimaging analysis versus CSF analysis is shown on Supplementray Table 2.

Regarding myelopathies: Spine CT imaging was analyzed in 8 dogs. The relative sensitivity value was 57.1% (95% CI: 18.4 to 90.1%) and specificity 25% (95% CI: 0.6 to 80.6%) for spine CT in the detection of changes in CSF inflammatory results. A comparison of results found in the spine CT neuroimaging analysis versus CSF analysis results is shown in Supplementray Table 3.

Overall, in the CT cases, the most common abnormality was multiple hypoattenuating or hyperattenuating lesions with or without contrast enlargement in the meninges and/or inside the lesions.

### 3.3. Neurological clinical signs

Of the 168 dogs, it was noted that the most common neurologic clinical signs were seizures. This included, in the presumptive MUO group, 56/152 (36.8%) of the cases and, in the ME infection group, 5/16 (31.3%) of the cases. This observation was followed by ataxia 37/152 (24.3%) in the MUO group and 3/16 (18.8%) in ME infection; complete neurological signs are presented in Supplementray Tables 4, 5.

Myoclonus was detected in ten dogs and, of those, six had CSF abnormal findings, five were positive for CDV, and one was positive for toxoplasmosis and neosporosis.

A total of 29/152 (17.3%) of dogs showed neurological clinical signs that suggested myelopathies, such as diffuse spine pain, neck pain, tetraparesis, and paraparesis.

## 4. Discussion

### 4.1. Prevalence of presumptive MUO and infectious ME

Presumptive MUO was significantly more common than infectious ME in Brazilian cases. Our findings corroborate a recent study in England that reported MUO (83.6%) as more common than infectious ME (16.4%) (17). In the study by Schwab et al. which utilized immunohistochemistry testing for 18 different infectious agents, an infectious etiology was found in 26% of the cases (18). Our investigation, using a neurologic PCR panel for 10 different infectious agents, found infectious agents in 9.52% of the cases. Infectious disease focused neurologic PCR tests were reported to be important to rule out infectious disease and have been suggested to be performed in cases with a suspicion of ME (2). Importantly, the current results demonstrated that normal CSF analysis does not rule out the possibility of a positive infectious disease PCR result. The present study additionally showed that 21.43% of patients with presumptive MUO had a normal result for CSF analysis which is also consistent with a previous publication (6).

Our study found, 4 agents were diagnosed by the PCR panel inclusive of CDV in 10 cases, toxoplasmosis in two cases, one case toxoplasmosis plus neosporosis, and borreliosis in three cases. This contrasts a recent study from England that reported more infectious ME caused by bacterial infections than CDV (17). CDV infection was reported to be associated with abnormal CSF analysis in 77.3% of dogs (19) which is similar to that observed in the present study (70%). As 10/16 (62.5%) cases were CDV positive in the current study, this supports that the agent remains common in Brazil.

### 4.2. Neuro imaging and CSF results

The investigation by Lamb et al. found abnormal MRI results in 76% of dogs with abnormal (inflammatory) results in the CSF analysis (20). Conversely, 24% of dogs with abnormal CSF results had normal brain MRI results which emphasizes that a normal brain MRI scan does not completely rule out the possibility of brain inflammatory disease (20). The present study corroborated the previous study showing abnormal brain MRI in 74% of cases with abnormal results in the CSF analysis whereas 26% of dogs with abnormal CSF results had normal brain MRI.

In the present investigation, samples from 22.7% of dogs with an abnormal brain and spinal cord MRI that was suggestive of a diagnosis of ME had a normal result in the CSF analysis. This finding is consistent with another study that showed that dogs with abnormal MRI suggestive of MUO could have normal results in the CSF analysis (6). To this point, it has been reported that the CSF cell count can be normal in 22% of MUO dogs (6).

The present study found that MRI is almost two times more sensitive than CT for finding intracranial inflammation in dogs with abnormal (inflammatory) CSF results. This information is consistent with a previous study which reported that MRI was more sensitive than CT for detecting lesions associated with intracranial inflammation in dogs (20, 21). Notably, an important limitation of CT imaging is that it produces a beam hardening artifact, more visible to adjacent petrous parts of the temporal bones, this artifact may affect the ability to interpret brainstem and cerebellar lesions (2). In Brazil, CT are more commonly performed than MRI. CT imaging has limited soft tissue detail and will miss some lesions that MRI will not. Nevertheless, if a CSF analysis is performed together with CT, it will increase the accuracy of obtaining a presumptive diagnosis of ME. CSF analysis has been reported as more sensitive than CT and MRI in identifying abnormalities consistent with inflammatory disease (2, 6). In a recent study, granulomas visualized in MUO cases also can “mimic” glioma on brain MRI and should not be ruled out from the differential diagnosis (22). The definitive diagnosis in MUO cases should be made with histopathology although it is acknowledged that this is also a challenging process (2, 6, 23).

Of all the MRI performed in CDV cases, 50% showed some abnormality, as small areas with hyperintensity in T2 and FLAIR. This corroborates previous MRI findings of CDV cases that showed demyelination signs hypersintensity in T2 and FLAIR images (22).

Regarding myelopathies, the present study showed that MRI is more sensitive than CT which is also consistent with previous studies that recommend MRI over CT for the detection of lesions in the spinal cord (6).

### 4.3. Neurological clinical signs

The present investigation included a higher proportion of younger dogs with ME which is consistent with a previous investigation that reported dogs aged between 3 and 7 years are most commonly affected by all subtypes of MUO (3). A second study of 40 cases demonstrated that MUO affected dogs with ages varying from 7.5 months to 9 years, median of 4 years at the time of initial presentation (14). In the present study, the Maltese was found the second most common breed affected by MUO. This breed was previously reported to have a higher MUO prevalence (14).

Seizure is the most common neurological clinical sign observed in the presumptive MUO and infectious ME groups. Seizure activity was consistent with that reported in a previous study in dogs with MUO (5, 14). Infectious with CDV also can cause neurological signs as central vestibular disease (head tilt, nystagmus, tendency to fall, cranial nerve and conscious proprioceptive deficits), cerebellar disease, and generalized or partial seizures (24). Myoclonus was not detected in all cases of CDV in the present investigation corroborating the Tipold et al. study, which demonstrated that myoclonus occurs in about 50% of these cases (24).

In a previous report, 8% of dogs diagnosed with MUO presented with neurological signs suggestive of myelopathy (6). There are few studies on MUO and myelopathies (25). The myelopathy could be localized anywhere in the spinal cord, and there were clinical signs ranging from general proprioceptive ataxia to paresis or paraplegia; spinal hyperesthesia was a common finding (6). In the present study, spinal cord signs were observed in 19% of the cases.

The definition of median survival time (MST) was beyond the scope of this investigation. This information has been previously discussed in the literature (6, 8). Likewise, nonspecific treatments for MUO or infectious ME were not evaluated or compared. Treatment options also have been discussed in the literature (8, 26, 27). However, most cases with MUO in the present investigation received a standard protocol suggested in the literature, using immunosuppressive doses of prednisolone and cytosine arasabine (26, 27). Several of these dogs showed an improvement in neurological signs and had a good quality of life for months or years, similar to what was described in recent literature (26, 27).

### 4.4. Limitations of this study

The results of this paper may be influenced by the study location which was inclusive of one private practice veterinary hospital in Brazil. In addition, the case review and imaging interpretation also used one examiner (FB) which could add bias. In addition, histopathology was not performed in order to confirm each diagnosis. That is, some lesions identified as consistent with MUO, could be reflective of another disease such as neoplasia or infectious granuloma. In addition, the sensitivity and specificity of the infectious disease PCR panel are unknown. Moreover, this panel is limited to 10 agents and thus is not inclusive of all infectious agents that can induce ME in dogs. Lastly, cultures were not performed on CSF samples.

## 5. Conclusion

The most common ME in this population of dogs was presumptive MUO, confirming our primary hypothesis. Testing with an infectious disease PCR panel is an important tool to exclude infectious ME, although, a larger PCR panel would be preferred. Normal CSF analysis does not preclude positive results in the PCR panel; however, a positive PCR panel usually accompanies an abnormal CSF analysis. MRI was more sensitive than CT to detect inflammatory changes in the brain and spinal cord, however, the use of CSF analysis is important to rule out inflammation even when MRI or CT are normal. An abnormal MRI or CT can be observed in the absence of abnormal CSF results. Additionally, the most common clinical signs in ME were seizures and age was not significantly different between groups with infectious ME and presumptive MUO cases.

## Data availability statement

The original contributions presented in the study are included in the article/Supplementary material, further inquiries can be directed to the corresponding author.

## Ethics statement

The animal study was approved by Reviewed by Ethics Committee of Pontifical Catholic University of Paraná (PUCPR, Brazil). The study was conducted in accordance with the local legislation and institutional requirements.

## Author contributions

FB conducted neurological examinations, magnetic resonances, image analysis, manuscript writing, results, and discussion. CC has improved English syntax and grammar and analysis of results and discussion. AB helped with data collection, manuscript writing and referencing, and organization. JV assisted in the correction of the manuscript. FM-F assisted in writing the manuscript, syntax, and analysis of results and discussion. All authors contributed to the article and approved the submitted version.

## References

[1] Radaelli ST Platt SR. Bacterial meningoencephalomyelitis in dogs: a retrospective study of 23 cases (1990-1999). J Vet Intern Med. (2002) 16:159–63. doi: 10.1892/0891-6640(2002)016<0159:bmidar>2.3.co;2, PMID: 11899031

[2] Coates JR Jeffery ND. Perspectives on meningoencephalomyelitis of unknown origin. Vet Clin Small Anim Pract. (2014) 44:1157–85. doi: 10.1016/j.cvsm.2014.07.009, PMID: 25239815

[3] Nessler JN Jo WK Osterhaus ADME Ludlow M Tipold A. Canine meningoencephalitis of unknown origin—the search for infectious agents in the cerebrospinal fluid via deep sequencing. Front Vet Sci. (2021) 8:645517. doi: 10.3389/fvets.2021.645517, PMID: 34950723 PMC8688736

[4] Talarico LR Schatzberg SJ. Idiopathic granulomatous and necrotising inflammatory disorders of the canine central nervous system: a review and future perspectives. J Small Anim Pract. (2010) 51:138–49. doi: 10.1111/j.1748-5827.2009.00823.x, PMID: 19814766

[5] O’sullivan S. The utility of immune profiling on formalin fixed nervous tissue to diagnose canine meningoencephalitis of unknown etiology. Guelph: University of Guelph (2021).

[6] Granger N Smith PM Jeffery ND. Clinical findings and treatment of non-infectious meningoencephalomyelitis in dogs: a systematic review of 457 published cases from 1962 to 2008. Vet J. (2010) 184:290–7. doi: 10.1016/j.tvjl.2009.03.031, PMID: 19410487

[7] Platt SR Olby NJ. BSAVA manual of canine and feline neurology. Quedgeley: British Small Animal Veterinary Association (2014).

[8] Lowrie M Smith PM Garosi L. Meningoencephalitis of unknown origin: investigation of prognostic factors and outcome using a standard treatment protocol. Vet Rec. (2013) 172:527. doi: 10.1136/vr.101431, PMID: 23462382

[9] Vitale S. DVM*, Kari Foss, immune-mediated central nervous system disease—Current knowledge and recommendations. Amsterdam: Elsevier Inc. (2018). 10.1053/j.tcam.2018.11.003 PMC7185457 30808493

[10] Zarfoss M Schatzberg S Venator K Cutter-Schatzberg K Cuddon P Pintar J. Combined cytosine arabinoside and prednisone therapy for meningoencephalitis of unknown aetiology in 10 dogs. J Small Anim Pract. (2006) 47:588–95. doi: 10.1111/j.1748-5827.2006.00172.x, PMID: 17004951

[11] Coates JR Barone G Dewey CW Vitale CL Holloway-Azene NM Sessions JK. Procarbazine as adjunctive therapy for treatment of dogs with presumptive antemortem diagnosis of granulomatous meningoencephalomyelitis: 21 cases (1998–2004). J Vet Intern Med. (2007) 21:100–6. doi: 10.1892/0891-6640(2007)21[100:paatft]2.0.co;2, PMID: 17338156

[12] Flegel T Boettcher IC Matiasek K Oevermann A Doherr MG Oechtering G. Comparison of oral administration of lomustine and prednisolone or prednisolone alone as treatment for granulomatous meningoencephalomyelitis or necrotizing encephalitis in dogs. J Am Vet Med Assoc. (2011) 238:337–45. doi: 10.2460/javma.238.3.337, PMID: 21281217

[13] Feliu-Pascual AL Matiasek K de Stefani A Beltrán E De Risio L. Efficacy of mycophenolate mofetil for the treatment of presumptive granulomatous meningoencephalomyelitis: preliminary results. 20th annual symposium of the European College of Veterinary Neurology Bern (Switzerland). (2007). 509.

[14] Wong MA Hopkins AL Meeks JC Clarke JD. Evaluation of treatment with a combination of azathioprine and prednisone in dogs with meningoencephalomyelitis of undetermined etiology: 40 cases (2000-2007). J Am Vet Med Assoc. (2010) 237:929–35. doi: 10.2460/javma.237.8.929, PMID: 20946080

[15] Bittencourt LHFB Pinto SIC. Prevalência das principais doenças infecciosas em cão e gato no hospital veterinário fag. Arquivos Brasileiros de Medicina Veterinária FAG. (2019) 2:73–87.

[16] Nicholas SJH Simon WJ. Small animal spinal disorders, diagnosis and surgery. 2nd ed. Amsterdam: Elsevier Ltd (1994).

[17] Gonçalves R De Decker S Walmsley G Butterfield S Maddox TW. Inflammatory disease affecting the central nervous system in dogs: a retrospective study in England (2010–2019). Front Vet Sci. (2022) 8:1638. doi: 10.3389/fvets.2021.819945 PMC8829331 35155652

[18] Schwab S Herden C Seeliger F Papaioannou N Psalla D Polizopulou Z. Non-suppurative meningoencephalitis of unknown origin in cats and dogs: an Immunohistochemical study. J Comp Pathol. (2007) 136:96–10. doi: 10.1016/j.jcpa.2006.11.006, PMID: 17275833 PMC7126569

[19] Tudury EA Arias MVB Bracarense APFL Megid J Dias Júnior RF. Clinical and laboratory findings in dogs with distemper encephalomyelitis. Ciência Rural. (1997) 27:229–35. doi: 10.1590/S0103-84781997000200010, PMID: 17084426

[20] Lamb CR Croson PJ Cappello R Cherubini GB. Magnetic resonance imaging findings in 25 dogs with inflammatory cerebrospinal fluid. Vet Radiol Ultrasound. (2005) 46:17–22. doi: 10.1111/j.1740-8261.2005.00003.x, PMID: 15693553

[21] Mathews VP Kuharik MA Edwards MK d’Amour PG Azzarelli B Dreesen RG. Gd-DTPA-enhanced MR imaging of experimental bacterial meningitis: evaluation and comparison with CT. Am J Roentgenol. (1989) 152:131–6. doi: 10.2214/ajr.152.1.131, PMID: 2783267

[22] Bathen-Noethen A Stein VM Puff C Baumgaertner W Tipold A. Magnetic resonance imaging findings in acute canine distemper virus infection. J Small Anim Pract. (2008) 49:460–7. doi: 10.1111/j.1748-5827.2008.00552.x, PMID: 18482329

[23] Diangelo L Cohen-Gadol A Heng HG Miller MA Hague DW Rossmeisl JH. Glioma mimics: magnetic resonance imaging characteristics of granulomas in dogs. Front Vet Sci. (2019) 28:286. doi: 10.3389/fvets.2019.00286 PMC6722480 31555671

[24] Tipold A Vandevelde M Jaggy A. Neurological manifestations of canine distemper virus infection. J Small Anim Pract. (1992) 33:466–70. doi: 10.1111/j.1748-5827.1992.tb01024.x, PMID: 35350433

[25] Cornelis I Volk HA Van Ham L De Decker S. Clinical presentation, diagnostic findings and outcome in dogs diagnosed with presumptive spinal-only meningoen-cephalomyelitis of unknown origin. J Small Anim Pract. (2017) 58:174–82. doi: 10.1111/jsap.12622, PMID: 28267222 PMC7166691

[26] Jeffery N Granger N. New insights into the treatment of meningoencephalomyelitis of unknown origin since 2009: a review of 671 cases. Front Vet Sci. (2023) 10:1114798. doi: 10.3389/fvets.2023.1114798 37008358 PMC10050685

[27] Beasley MJ Shores A. Perspectives on pharmacologic strategies in the Management of Meningoencephalomyelitis of unknown origin in dogs. Front Vet Sci. (2023) 10:577. doi: 10.3389/fvets.2023.1167002 PMC10205981 37234070

