# Supplementary material for: A comparison between neurological clinical signs, cerebrospinal fluid analysis, cross-sectional CNS imaging, and infectious disease testing in 168 dogs with infectious or immune-mediated meningoencephalomyelitis from Brazil
Source: Front Vet Sci. 2023 Oct 25;10:1239106. doi: 10.3389/fvets.2023.1239106 (PMC10630916; doi:10.3389/fvets.2023.1239106)
Supplement: Supplementary file 1 [file Table_1.docx]

**Supplementary Table 1-** Representation of canine breeds present in the study.

| **Breed** | **Frequency** | **Percentage %** |
| --- | --- | --- |
| Mixed breed | 47 | 28% |
| Maltese | 22 | 13.1% |
| Yorkshire Terrier | 11 | 6.5% |
| Lhasa Apso | 10 | 5.9% |
| French Bulldog | 9 | 5.4% |
| German Sptiz | 9 | 5.4% |
| Shih tzu | 8 | 4.8% |
| Pinscher | 7 | 4.1% |
| Pug | 7 | 4.1% |
| Poodle | 6 | 3.5% |
| Schnauzer | 4 | 2.4% |
| Chihuahua | 3 | 1.8% |
| Golden Retriever | 3 | 1.8% |
| Labrador Retriever | 3 | 1.8% |
| Beagle | 2 | 1.2% |
| Border Collie | 2 | 1.2% |
| Brazillian Terrier | 2 | 1.2% |
| Pomeranian Lulu | 1 | 0.6% |
| Akita | 1 | 0.6% |
| American Staffordshire Terrier | 1 | 0.6% |
| Boxer | 1 | 0.6% |
| English Cocker Spaniel | 1 | 0.6% |
| Dachshund | 1 | 0.6% |
| Siberian Husky | 1 | 0.6% |
| Pekingese | 1 | 0.6% |
| American Pitbull Terrier | 1 | 0.6% |
| Pointer | 1 | 0.6% |
| Rottweiller | 1 | 0.6% |
| Shar pei | 1 | 0.6% |
| Newfoundland | 1 | 0.6% |
| **Total** | **168** | **100%** |
