# Supplementary material for: A comparison between neurological clinical signs, cerebrospinal fluid analysis, cross-sectional CNS imaging, and infectious disease testing in 168 dogs with infectious or immune-mediated meningoencephalomyelitis from Brazil
Source: Front Vet Sci. 2023 Oct 25;10:1239106. doi: 10.3389/fvets.2023.1239106 (PMC10630916; doi:10.3389/fvets.2023.1239106)
Supplement: Supplementary file 2 [file Table_2.docx]

**Supplementary Table 2-** Comparison of brain neuroimaging results to the CSF analysis.

| **NEUROIMAGING COMPARISON** | **MRI/CT** | **CSF** | **%** |
| --- | --- | --- | --- |
| MRI normal x CSF normal | 16 | 25 | 64.00% |
| MRI normal x CSF abnormal | 21 | 81 | 25.92% |
| MRI abnormal x CSF normal | 9 | 25 | 36.00% |
| MRI abnormal x CSF abnormal | 60 | 81 | 74.08% |
| CT normal x CSF normal | 5 | 7 | 71.43% |
| CT normal x CSF abnormal | 14 | 23 | 60.87% |
| CT abnormal x CSF normal | 2 | 7 | 28.57% |
| CT abnormal x CSF abnormal | 9 | 23 | 39.13% |
