# Supplementary material for: A comparison between neurological clinical signs, cerebrospinal fluid analysis, cross-sectional CNS imaging, and infectious disease testing in 168 dogs with infectious or immune-mediated meningoencephalomyelitis from Brazil
Source: Front Vet Sci. 2023 Oct 25;10:1239106. doi: 10.3389/fvets.2023.1239106 (PMC10630916; doi:10.3389/fvets.2023.1239106)
Supplement: Supplementary file 3 [file Table_3.docx]

**Supplementary Table 3-** Comparison of spinal cord neuroimaging results to the CSF analysis.

| **NEUROIMAGING COMPARISON** | **MRI/CT** | **CSF** | **%** |
| --- | --- | --- | --- |
| MRI normal x CSF normal | 2 | 3 | 66.66% |
| MRI normal x CSF abnormal | 2 | 21 | 9.52% |
| MRI abnormal x CSF normal | 1 | 3 | 33.34% |
| MRI abnormal x CSF abnormal | 19 | 21 | 90.48% |
| CT normal x CSF normal | 1 | 1 | 100% |
| CT normal x CSF abnormal | 3 | 7 | 42.86% |
| CT abnormal x CSF normal | 0 | 1 | 0% |
| CT abnormal x CSF abnormal | 4 | 7 | 57.14% |
