# Supplementary material for: A comparison between neurological clinical signs, cerebrospinal fluid analysis, cross-sectional CNS imaging, and infectious disease testing in 168 dogs with infectious or immune-mediated meningoencephalomyelitis from Brazil
Source: Front Vet Sci. 2023 Oct 25;10:1239106. doi: 10.3389/fvets.2023.1239106 (PMC10630916; doi:10.3389/fvets.2023.1239106)
Supplement: Supplementary file 4 [file Table_4.docx]

**Supplementary Table 4-** Clinical signs of dogs with presumptive MUO

| **Neurological signs** | **Frequency** | **Percentage (%)** |
| --- | --- | --- |
| Seizure | 56 | 36.84 |
| Ataxia | 37 | 24.34 |
| Neck pain | 12 | 7.89 |
| Myoclonus | 7 | 4.61 |
| Trigemial nerve paralysis | 7 | 4.61 |
| Diffuse spinal pain | 7 | 4.60 |
| Myoclonus | 6 | 3.95 |
| Proprioception deficits | 6 | 3.95 |
| Mental status changes | 5 | 3.29 |
| Paraparesis | 4 | 2.63 |
| Tetraparesis | 3 | 1.97 |
| Hemiparesis | 2 | 1.32 |
| **Total** | **152** | **100%** |
