# Supplementary material for: A comparison between neurological clinical signs, cerebrospinal fluid analysis, cross-sectional CNS imaging, and infectious disease testing in 168 dogs with infectious or immune-mediated meningoencephalomyelitis from Brazil
Source: Front Vet Sci. 2023 Oct 25;10:1239106. doi: 10.3389/fvets.2023.1239106 (PMC10630916; doi:10.3389/fvets.2023.1239106)
Supplement: Supplementary file 5 [file Table_5.docx]

**Supplementary Table 5-** Clinical signs of dogs ME positive for infectious diseases

| **Neurology clinical signs** | **Frequency** | **Percentage (%)** |
| --- | --- | --- |
| Seizure | 5 | 31.25 |
| Ataxia | 3 | 18.75 |
| Myoclonus | 3 | 18.75 |
| Neck pain | 3 | 18.75 |
| Visual deficit | 2 | 12.50 |
| **Total** | **16** | **100%** |
